# Serum BDNF and GDNF in Chinese male patients with deficit schizophrenia and their relationships with neurocognitive dysfunction

**DOI:** 10.1186/s12888-019-2231-3

**Published:** 2019-08-16

**Authors:** Xiaowei Tang, Chao Zhou, Ju Gao, Weiwei Duan, Miao Yu, Wenhuan Xiao, Xiaobin Zhang, Hui Dong, Xiang Wang, Xiangrong Zhang

**Affiliations:** 1 Department of Geriatric Psychiatry, Affiliated Nanjing Brain Hospital, Nanjing Medical University, No. 264 Guangzhou Road, Nanjing, Jiangsu Province, 210029 People’s Republic of China; 2 Affiliated WuTaiShan Hospital of Medical College of Yangzhou University, Yangzhou, 225003 Jiangsu China; 3 Shanghai Changning Mental Health Center, Shanghai, 210029 China; 4 Jining Medical University, Jining, 272067 Shandong China; 5 Medical Psychological Institute of the Second Xiangya Hospital, Central South University, Changsha, 410011 Hunan China

**Keywords:** Schizophrenia, Deficit schizophrenia, Brain-derived neurotrophic factor, Glial cell line-derived neurotrophic factor, Neurocognition

## Abstract

**Background:**

To measure the serum levels of brain-derived neurotrophic factor (BDNF) and glial cell line-derived neurotrophic factor (GDNF) in deficit schizophrenia (DS), in order to examine the association between these two neurotrophic factors (NFs) and cognitive performance.

**Methods:**

A total of 109 male patients [51 DS and 58 non-deficit schizophrenia (NDS)] with schizophrenia and 40 sex and age matched healthy controls (HC) participated in this study. Processing speed, attention, executive function, and working memory of all subjects were assessed by means of a battery of classical neuropsychological tests. Serum BDNF and GDNF levels were measured simultaneously using a double-antibody sandwich enzyme-linked immunosorbent assay (ELISA).

**Results:**

There were significant differences in the overall cognitive test scores between three groups (all *p* < 0.001). Serum BDNF levels were significantly lower in patients (DS and NDS) than in HC (*p* < 0.001). Furthermore, BDNF levels were lower in the DS compared to the NDS group, although not significantly. However, there was no difference in the GDNF levels between patients (DS and NDS) and HC. GDNF levels were positively correlated with scores of **Stroop** words only (*r* = 0.311, *p* = 0.033), Stroop colors only (*r* = 0.356, *p* = 0.014) and Stroop interference (*r* = 0.348, *p* = 0.016) in DS group**.**

**Conclusion:**

Serum BDNF may be an unsuitable biomarker for DS, despite a significant decrease in schizophrenia patients. The different neurocognitive performance between the DS and NDS patients indicates that DS may be a separate clinical entity of schizophrenia. Finally, higher serum GDNF levels are associated with better cognitive performance in DS patients, indicating a possible neuroprotective function in DS.

## Background

Schizophrenia is a debilitating psychiatric disorder resulting from abnormal brain development. Previous studies suggest that the etiology of schizophrenia is a combination of genetics and environmental factors, which lead to mal-development of the central nervous system (CNS) and impaired neurotransmission [1, 2]. Neurodevelopmental aberrations influenced by neurotrophic factors (NFs) are important paradigms in understanding schizophrenia pathogenesis.

Brain-derived neurotrophic factor (BDNF), as one of NFs, is thought to be the key in the development and maintenance of cortical neurons and synapses. BDNF is widely distributed in the CNS and plays an important role in the survival, differentiation and growth of neurons during the developmental stages in neonatal individuals, synaptic plasticity and behavior in adulthood [3, 4]. Many evidences suggest that BDNF is involved in the pathophysiological process of schizophrenia [5]. Previous studies have shown that peripheral BDNF in patients with chronic or first-episode schizophrenia are decreased, while BDNF mRNA was reduced in the postmortem brain of patients [6–8]. However, other reports have shown inconsistent results [9]. BDNF is also associated with learning and memory, as demonstrated in gene knockout animal model [10]. Schizophrenics have a wide range of cognitive deficits, such as learning, memory, executive function and attention. Animal models and clinical trials have also shown that BDNF levels are positively correlated with cognitive impairment [8, 11–13]. Glial cell **line-**derived neurotrophic factor (GDNF) is one of the most potent trophic factors for dopaminergic neurons of the mammalian CNS. The most prominent feature of GDNF is its ability to support the survival of dopaminergic and motorneurons. GDNF heterozygous mutant mice showed the impaired water-maze learning performance, indicating its role in cognitive abilities [14]. Therefore, GDNF may be potentially relevant to the dopaminergic and neurodevelopmental hypothesis of schizophrenia. Four studies published so far on GDNF serum levels in schizophrenia [8, 15–17] have shown inconsistent results, two of which suggested that serum GDNF levels were associated with cognitive function [8, 15]. Considering these conflicting results, the relationship between NFs and schizophrenia needs to be studied further. The inconsistency of previous studies on the association of BDNF and GDNF with schizophrenia may be related to the confounding effects of the severity of symptoms, age, gender, sample population, different stages of illness and medication history. However, since schizophrenia is a complex and heterogeneous disorder, studying specific clinical phenotypes rather than the whole spectrum would be more beneficial to better understand the disease.

Since Carpenter described deficit schizophrenia (DS) in a homogeneous subclass of schizophrenia patients characterized by primary and continuous negative symptoms, a large number of studies have shown that the deficit and non-deficit forms of schizophrenia (DS and NDS) differ in several parameters, such as biological correlates, risk factors, etiological factors and treatment response [18]. There is mounting evidences that DS may be a distinct subtype of schizophrenia, which may help to reduce the heterogeneity of this disease. Akyol et al. reported that the serum BDNF levels in DS were significantly lower than those in the healthy individuals, while those in NDS patients were similar to normal levels [19]. In contrast, Valiente-Gómez et al. did not find any significant differences across the three groups [20]. To our best knowledge, there is no report on serum levels of GDNF in DS patients, neither literatures on the association between serum BDNF or GDNF concentrations and cognitive dysfunction in DS patients.

Although there have been inconsistencies in the studies that investigated the BDNF and GDNF levels in patients with schizophrenia; the majority of the studies revealed that these two NFs are closely related to the pathological process of this disease [7–9, 15–17]. At present, the research on BDNF levels in DS patients is also inconsistent. Therefore, the present study aimed to probe whether there is a discrepancy between patients with DS, NDS, and healthy controls (HC) in terms of serum BDNF and GDNF levels. Meanwhile, we would like to evaluate the relationship between BDNF or GDNF levels and cognitive impairment in DS.

## Methods

### Participants

A total of 109 consecutive male patients with clinically stable schizophrenia from the Wutaishan Hospital of Yangzhou, Jiangsu Province, and 40 sex and age matched HC were recruited for this study. All patients met the Diagnostic Interview and the Diagnostic and Statistical Manual-IV (DSM-IV) criteria for schizophrenia, which was confirmed by two independent senior psychiatrists on the basis of the Structured Clinical Interview for DSM-IV (SCID). In addition, the patients had the chronic course of disease at least 5 years, and had received stable doses of oral antipsychotic drugs for at least 12 months before recruitment. As the patients were prescribed different antipsychotic drugs, all drugs were converted into chlorpromazine equivalents in order to compare the medium dose of antipsychotics between DS and NDS groups. DS diagnosis was confirmed based on the Chinese version of the Schedule for Deficit Syndrome (SDS-C) [21]. The enrolled patients were divided into 51 DS and 58 NDS based on the SDS-C. Exclusion criteria included a history of head injury, neurological disorders, dementia, mental retardation or significantly impaired vision and red-green colorblindness, comorbidities involving major organs, alcohol/substance abuse or dependence, and electroconvulsive therapy.

Healthy controls with no personal or family history of psychiatric disorders were recruited through advertisements in the local community. None of them had any history of drug and alcohol abuse/dependence, nor significantly impaired vision and red-green colorblindness. All subjects were Han Chinese aged between 30 and 60 years, and were assessed in terms of weight and height in order to calculate their body mass index (BMI). The study protocol was approved by the ethical review committee of the local hospital, and all subjects participated after giving written informed consent.

### Assessment of clinical symptoms

Two psychiatrists with minimum 5 years’ experience in clinical practice assessed the psychopathology of the patients using the Brief Psychiatric Rating Scale (BPRS), the Scale for the Assessment of Negative Symptoms (SANS), and the Scale for the Assessment of Positive Symptoms (SAPS). To ensure consistency and reliability of ratings across the participants, the two psychiatrists attended a training session on these clinical scales prior to the study. The 18-item BPRS was classified into positive, negative, disorganized, and affect symptom subscores based on the findings of the most comprehensive factor analysis [22].

### Neurocognitive assessments

Processing speed, attention, executive function, and working memory were assessed by classical neuropsychological tests like Digit Vigilance test (DVT), Verbal Fluency tests (VFT-animals and VFT-actions), Stroop Color-Word test (SCWT), Block Design (Wechsler adult intelligence scale-Chinese Revision WAIS-RC), and Paced Auditory Serial Addition Test (PASAT). The cognitive functions of all subjects were examined by two experienced psychiatrics in a fixed test laboratory.

### Measurement of serum BDNF and GDNF levels

Serum samples from all participants were collected between 7 and 9 a.m. at the same time following an overnight fast, and stored at − 80 °C till analysis. Serum BDNF and GDNF levels were measured by ELISA according to the manufacturer’s instructions (Promega, Madison, WI, USA). Each sample was tested twice for BDNF and GDNF levels and the mean of two measurements were used for statistical analysis. Inter- and intra-assay variation coefficients of each NF were less than 5%.

### Statistical analysis

All data were analyzed using the Statistical Package for Social Sciences (SPSS) version 13.0. Test scores are presented as means with standard deviations. Categorical variables were analyzed by the chi-square test. Probability values were based on two-samples t-tests or one-way analysis of variance (ANOVA) for continuous variables. Bonferroni test was used as the post hoc test of ANOVA. There were significant differences of education duration among three groups, therefore education duration was performed as a covariate in the comparison of neuropsychological performances and NFs levels by the analysis of covariance (ANCOVA) with post hoc Bonferroni correction as appropriate. When comparing the cognitive performance and NFs levels between the DS and NDS group, the BPRS negative symptoms and SANS scores were performed as a covariate because they were higher in the DS group than in the NDS group. Correlation analysis was performed by partial correlation test. For all analyses, the significance level (*p* value) was set at 0.05.

## Results

### Sample characteristics

As shown in Table 1, no significant differences were present between the patients and HC in terms of age and BMI, except for education (*p =* 0.023). There were also no significant differences in the age of onset, duration of illness, and smoking ratio between the two patient groups. In addition, their antipsychotic medication doses were similar when converted to equivalent doses of chlorpromazine [23]. As expected, the DS patients showed more severe total and negative psychopathological symptoms (all *p* < 0.001) than the NDS patients, but both groups had similar positive, affect or disorganized symptoms (all *p* > 0.05). None of the patients were receiving antidepressant or mood-stabilizing drugs.

**Table 1:** Demographic and clinical characteristics of DS and NDS patients and healthy controls

	Deficit schizophrenia (DS; *N* = 51)	Nondeficit schizophrenia (NDS; *N* = 58)	Healthy controls (HC; *N* = 40)	*F/t/χ2*	*p* Value
Age (years)	50.24 ± 6.85	47.90 ± 6.84	46.78 ± 10.73	2.245	0.110
Education (years)	8.71 ± 2.44	8.79 ± 2.19	10.08 ± 3.21	3.857	0.023
BMI (kg/m^2^)	25.10 ± 1.28	25.21 ± 1.17	24.82 ± 1.16	1.212	0.301
Age of Onset (years)	22.02 ± 2.75	22.16 ± 2.91	–	−0.249	0.804
Duration of illness(years)	28.22 ± 6.13	25.74 ± 6.94	–	1.962	0.052
Chlorpromazine eguivalents (mg)	462.65 ± 238.61	524.48 ± 211.00	–	−1.436	0.154
Smoking ratio (%)	54.90	69.00	–	2.287	0.130
BPRS score					
Positive symptoms	6.35 ± 1.16	6.45 ± 1.20	–	−0.420	0.675
Negative symptoms	12.43 ± 1.78	7.47 ± 1.25	–	17.028	< 0.001
Disorganized symptoms	6.75 ± 1.57	6.59 ± 1.04	–	0.628	0.531
Affect syndromes	6.67 ± 1.11	7.02 ± 1.32	–	−1.492	0.139
Sum	32.18 ± 3.17	27.52 ± 2.86	–	8.070	< 0.001
SAPS total score	9.76 ± 3.84	10.31 ± 4.69	–	−0.659	0.511
SANS total score	57.41 ± 9.30	32.62 ± 7.51	–	15.385	< 0.001

*BMI* body mass index, *BPRS* brief psychiatric rating scale, *SAPS* the Scale for the Assessment of Positive Symptoms, *SANS* the Scale for the Assessment of Negative Symptoms

### Cognitive performance in schizophrenia and HC

The performance of cognitive tests is summarized in Table 2. The inter-group differences were analyzed by ANOVA, which indicated significant differences in the overall scores (all *p* < 0.001). Post hoc analysis revealed that the DS group performed significantly worse than the HC as well as the NDS group in terms of overall cognitive test scores. The NDS group also performed significantly worse than the HC group in all cognitive tests (all *p* < 0.01), except for DVT (*p* > 0.05).

**Table 2:** Comparison of neuropsychological performances among DS and NDS patients and healthy controls

	Deficit schizophrenia (DS,*N* = 51)	Nondeficit schizophrenia (NDS,*N* = 58)	Healthy controls (HC,*N* = 40)	F	*p* Value
DVT (seconds)	347.14 ± 238.41^**^	203.10 ± 93.88^##^	133.31 ± 45.66	22.553	< 0.001
Stroop words only	40.67 ± 20.13^**^	58.16 ± 18.22^##^	79.03 ± 17.13^△△^	42.790	< 0.001
Stroop colors only	25.37 ± 12.49^**^	36.00 ± 12.03^##^	50.68 ± 17.21^△△^	34.160	< 0.001
Stroop interference	16.67 ± 9.79^**^	22.59 ± 8.67^##^	30.60 ± 10.91^△△^	19.982	< 0.001
WAIS-RS(Block Design)	13.67 ± 8.70^**^	20.90 ± 6.84^##^	33.18 ± 7.02^△△^	68.015	< 0.001
PASAT correct	15.55 ± 8.05^**^	26.26 ± 8.51^##^	34.68 ± 10.63^△△^	47.827	< 0.001
PASAT try	17.82 ± 9.04^**^	30.24 ± 8.77^##^	38.28 ± 9.93^△△^	54.236	< 0.001
Category fluency score	14.45 ± 5.14^**^	19.14 ± 7.69^##^	29.80 ± 9.15^△△^	44.002	< 0.001

^**^p < 0.001 DS vs. HC; ^##^*p* < 0.001 DS vs. NDS; ^△△^*p* < 0.001 NDS vs. HC

### Serum BDNF and GDNF in schizophrenia and HC

**Fig.1:**
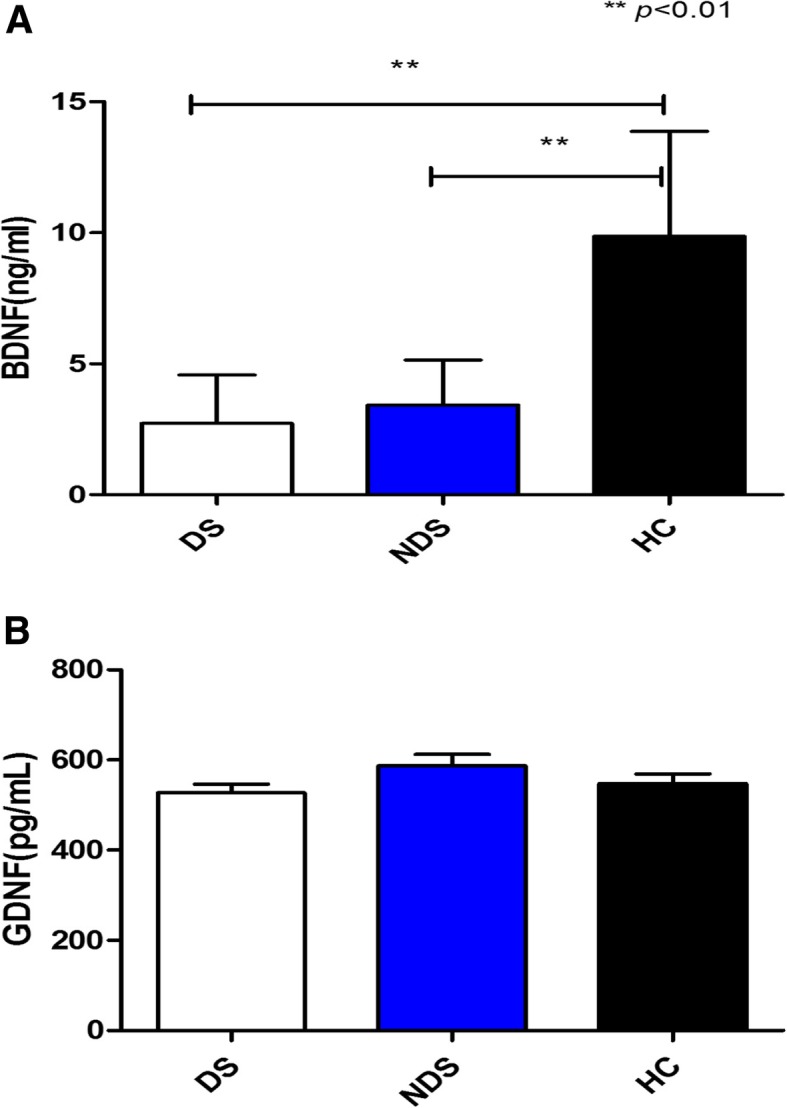
**a** Serum BDNF levels in DS and NDS patients and HC. **b** Serum GDNF levels in DS and NDS patients and HC. **a** shows that serum BDNF levels were significantly lower in the patients (DS:2.72 ± 1.85 ng/ml, NDS: 3.43 ± 1.71 ng/ml) compared to the HC (9.86 ± 4.01 ng/ml) (*F* = 96.456, *p* < 0.001). But there is no difference in the BDNF levels between DS and NDS (*p* > 0.05). **b** shows that there is no difference in the GDNF levels of the patients (DS and NDS) and HC (527.32 ± 136.86 pg/ml vs. 587.03 ± 193.90 pg/ml vs. 547.49 ± 134.66 pg/ml, *F* = 1.992, *p* = 0.140)

Serum BDNF levels were significantly lower in the patients compared to the HC (2.72 ± 1.85 ng/ml vs. 3.43 ± 1.71 ng/ml vs. 9.86 ± 4.01 ng/ml, *F* = 96.456, *p* < 0.001)(Fig. 1a). Furthermore, BDNF levels showed a tendency of decrease in the DS group than the NDS group, but without statistical significance (*p >* 0.05). There was no significant difference in the GDNF levels of the patients (DS and NDS) and HC (527.32 ± 136.86 pg/ml vs. 587.03 ± 193.90 pg/ml vs. 547.49 ± 134.66 pg/ml, *F* = 1.992, *p* = 0.140) (Fig. 1b).

### The relationships between BDNF or GDNF levels and cognitive functioning

**Fig.2:**
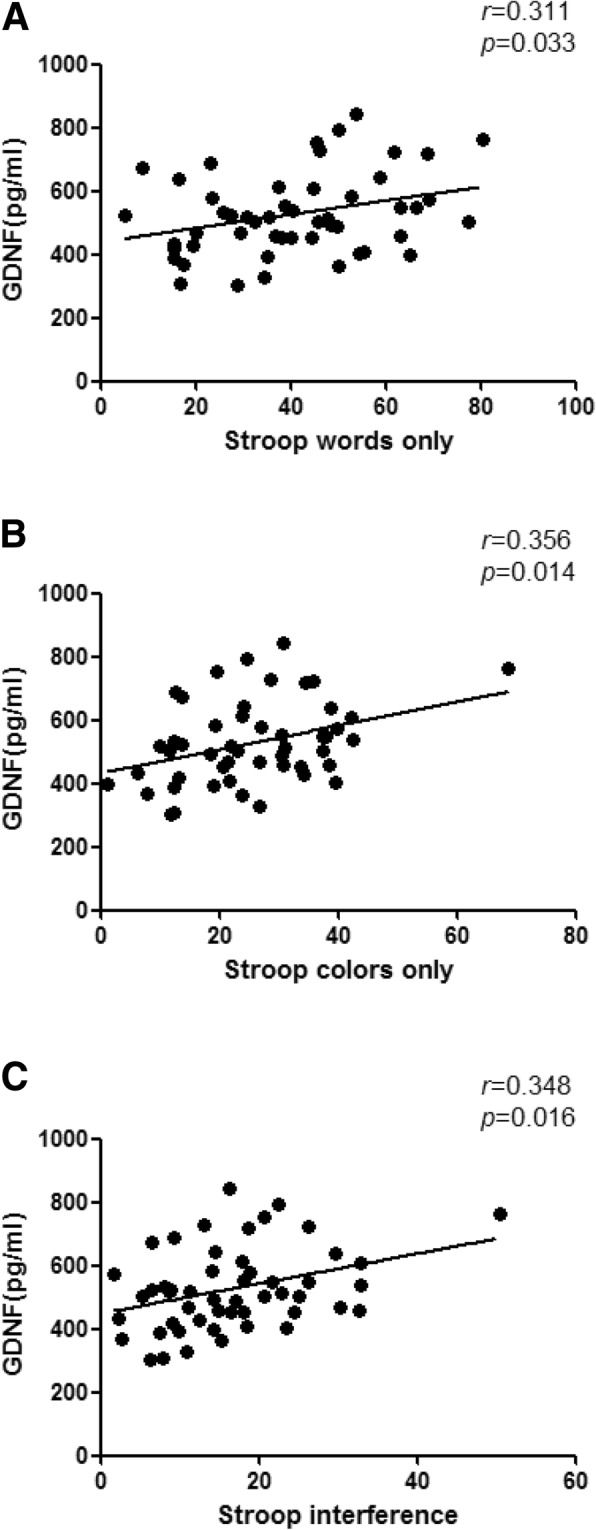
**a** Correlation between serum GDNF levels and the Stroop words in DS. **b** Correlation between serum GDNF levels and the Stroop colors in DS. **c** Correlation between serum GDNF levels and the Stroop interference in DS. **a**, **b** and **c** shows that GDNF levels were positively correlated with scores of Stroop words only (*r* = 0.311, *p* = 0.033), Stroop colors only (*r* = 0.356, *p* = 0.014) and Stroop interference (*r* = 0.348, *p* = 0.016) in DS group

GDNF levels were positively correlated with scores of Stroop words only (*r* = 0.311, *p* = 0.033), Stroop colors only (*r* = 0.356, *p* = 0.014) and Stroop interference (*r* = 0.348, *p* = 0.016) in DS group (Fig. 2a, b and c). There were no correlations between either BDNF or GDNF levels and the cognitive tests in NDS and HC groups.

## Discussion

Our study for the first time detected serum BDNF and GDNF levels in patients with DS and NDS in Chinese population [24]. Given that serum BDNF levels might be influenced by the menstrual cycle, we only chose male individuals as research subjects [25]. It has been reported that the peripheral levels of BDNF can reflect the changes of CNS levels [26]. A large amount of evidence showed BDNF may be related to the pathophysiology of schizophrenia because of its role in the development, regeneration, survival and maintenance of brain neurons [9, 27]. Some literatures reported that the levels of BDNF and TrkB receptor mRNA in prefrontal cortex and hippocampus of patients with chronic schizophrenia were significantly decreased [28, 29]. Our study also found a significant reduction of serum BDNF in the chronic schizophrenic patients (DS and NDS) compared to HC, which is consistent with many previous studies [12, 30]. However, no significant differences in serum BDNF were seen between the DS and NDS subgroups, while only a slight decrease was found in the DS patients. This is also consistent with a previous study showing decreased serum BNDF in the schizophrenic patients compared to the healthy individuals, without any significant difference between the DS and NDS subgroups [20]. In another study, Akyol et al. reported reduced serum BDNF levels in the DS patients compared to the controls but no significant difference between the NDS and control groups [19]. These contradictory findings may be attributed to confounding factors like the patients’ age, gender, severity of the illness, treatment modules, and the genetic background of the patients. Notably, both male and female schizophrenic patients were included in Akyol’s study.

To the best of our knowledge, this is the first study to explore the serum GDNF levels in DS patients. However, we didn’t find any significant differences among the DS, NDS and HC groups in terms of serum GDNF, which is consistent with one recent study that reported similar serum GDNF levels in the chronic schizophrenia patients and controls [15]. Another study also showed no differences in serum GDNF between female schizophrenia patients and healthy individuals [16]. Our previous study have demonstrated that serum GDNF were significantly lower in drug-free schizophrenic patients than in healthy controls, and the reduced serum GDNF gradually returned to normal levels with the improvement in psychiatric symptoms following antipsychotic therapy [31]. Increase in GDNF levels during treatment is in line with a few previous studies that showed that antipsychotic medications stimulated C6 glioma cells to secrete GDNF [32], and enhanced GDNF signaling in experimental [33] and clinical settings [34]. In our study, the chronic schizophrenia patients had received long-term antipsychotic treatment during hospitalization, which likely altered their serum GDNF levels to near normal levels. Therefore, more detailed longitudinal studies are required to determine if GDNF may be associated with the pathogenesis of schizophrenia and the response to pharmacological treatment. In contrast, Tunca et al. reported lower serum GDNF levels in schizophrenic inpatients or outpatients who received antipsychotic and antidepressant treatment [17]. The likely reasons for this discrepancy could be the different ages and antipsychotic drug doses in the two studies.

Another major finding of our study is the significant difference in the cognitive scores, except that for DVT, seen in the pairwise comparisons between DS, NDS and HC groups. These findings are consistent with that of our previous study indicating greater impairments in every neuropsychological measure and cognitive domain in the DS compared to the NDS patients [35]. The present study provided evidence to support the hypothesis that the deficit syndrome may be a specific subgroup within schizophrenia. In addition, this is the first study to correlate BDNF and GDNF levels with cognitive dysfunction in DS and NDS patients. Consistent with one study reporting no correlation between the serum levels of BDNF and cognitive functions in schizophrenia patients [36], we also found that BDNF serum levels were not related to cognitive function in DS, NDS, and HC groups. However, the reported BDNF levels in healthy controls were negatively correlated with verbal working memory in this article. In a recent study of a Chinese sample, the authors found that there was no correlation between serum BDNF levels and cognitive function in patients with first-episode schizophrenia and healthy controls [37]. Our result contradicts previous studies on chronic schizophrenia patients [12, 13] and a recent meta-analysis [5], which suggested that higher BDNF expression was associated with better neurocognitive performance in schizophrenia patients. Several factors can account for this discrepancy, such as different cognitive tests used, the duration of the illness, comorbidities, smoking, diet, obesity and even genetic background of the patients.

The third major finding of our study is that higher serum levels of GDNF are associated with better performance on Stroop words only, Stroop colors only and Stroop interference in the DS group. However, no significant correlations were observed between GDNF and cognitive test scores in either the NDS or HC groups. Stroop interference test is used to evaluate cognitive flexibility which is one of the critical roles of executive function domain, whereas Stroop words only and Stroop colors only test provides information on sustained attention. Our results indicate that lower GDNF serum levels in DS patients are related to more severe cognitive impairments in executive function and attention. GDNF is widely expressed in the brain and play particularly important roles in the physiology of catecholaminergic neurons [38]. Moreover, it has been reported to be related to the development and maintenance of various types of neurons: dopaminergic, serotonergic, noradrenergic and GABAergic [39], which are all involved in the pathological process of schizophrenia. Previous animal studies demonstrated that GDNF was closely related to learning and memory [14, 40] and GDNF+/− mutant mice exhibit abnormal hippocampal synaptic transmission [41], indicative of a role for GDNF in cognitive abilities. These results may explain, to some extent, the association between serum GDNF with several subsets of cognitive abilities in DS patients in our study. However, one study has shown that higher GDNF levels result in more severe attention deficit in schizophrenia outpatients [15]. But we found that lower serum levels of GDNF were associated with more severe cognitive impairment in attention distribution and verbal fluency in first-episode schizophrenics in our previous study [8]. The differences of sample sources, subject characteristics, and study paradigms in these studies may be one of the reasons for the inconsistent findings.

### Limitations of the study

Our study has several limitations. First, BDNF contains multiple proteolytic isoforms (pro-BDNF, truncated BDNF, mature BDNF) in the serum, but we only tested serum total BDNF levels. Carlino et al. confirmed that mature BDNF and pro-BDNF levels were increased while that of truncated BDNF was decreased in the schizophrenic patients, and low serum levels of the truncated BDNF isoform were associated with cognitive impairments [42]. Therefore, the proteolytic isoforms of BDNF may affect the results of the present study. Second, we did not analyze the influence of inflammation on the correlation between BDNF and schizophrenia. Some studies reported that interactions between inflammatory factors and BDNF may be implicated in the pathophysiology and cognitive impairment seen in chronic schizophrenia [43, 44]. Therefore, an as yet unknown interaction between inflammatory factors and BDNF in DS may be an avenue worth researching further. Third, it is unclear whether GDNF content in serum is correlated with the brain GDNF content. Previous studies have found that GDNF had a very low capacity of crossing the blood-brain barrier [45]. Fourth, this study is an exploratory analysis of a relatively small sample, but did not include correcting the correlations between NFs and cognitive performance for multiple comparisons. We attempt to increase the homogeneity of subjects by eliminating or minimizing confounding factors, such as gender, fluctuations of psychiatric symptoms and social environment, which will make it difficult to recruit DS and NDS patients. The present study included larger DS samples, compared with other published studies of NFs in this group. However, the limited power to be the current sample size means that we cannot accurately determine the different contributions of cognitive impairment and subgroup effects on NFs alterations. A larger sample size is needed to increase the statistical power in future research. Finally, all patients in our study had been receiving medications, and their effect on NFs could not be ruled out. In addition, as we only recruited male subjects, the future researches include both male and female participants would be needed.

## Conclusions

In conclusion, our preliminary data suggests that serum BDNF level maybe not a suitable biomarker for DS, despite its significantly lower levels in the schizophrenia patients. In addition, the differences in the neurocognitive functions of DS and NDS patients support the concept of DS being a different clinical entity of schizophrenia. Although serum GDNF levels in the DS group were not significantly different from the NDS and HC groups, only the DS patients showed an association between higher serum GDNF levels with better cognitive performance, which indicates that GDNF may be a protective factor in maintaining cognitive function in DS. Therefore, future longitudinal studies that include untreated patients with DS would provide new insights.

## Data Availability

The de-identified dataset used and/or analysed during the current study are available from the corresponding author on reasonable request.

## Abbreviations

ANOVA: Analysis of variance
BDNF: Brain-derived neurotrophic factor
BMI: Body mass index
BPRS: Brief psychiatric rating scale
CNS: Central nervous system
DS: Deficit schizophrenia
DSM-IV: Diagnostic and statistical manual-IV
DVT: Digit Vigilance test
ELISA: Enzyme-linked immunosorbent assay
GDNF: Glial cell line-derived neurotrophic factor
NDS: Non-deficit schizophrenia
NFs: Neurotrophic factors
PASAT: Paced auditory serial addition test
SANS: Scale for the assessment of negative symptoms
SAPS: Scale for the assessment of positive symptoms
SCID: Structured clinical interview for DSM-IV
SCWT: Stroop color-word test
SDS-C: Chinese version of the schedule for deficit syndrome
SPSS: Statistical package for social sciences
VFT: Verbal fluency tests
WAIS-RC: Wechsler adult intelligence scale-Chinese Revision

## References

[1] Lewis DA, Gonzalez-Burgos G (2006). Pathophysiologically based treatment interventions in schizophrenia. Nat Med.

[2] Stephan KE, Baldeweg T, Friston KJ (2006). Synaptic plasticity and dysconnection in schizophrenia. Biol Psychiatry.

[3] Lu B (2003). BDNF and activity-dependent synaptic modulation. Learn Mem.

[4] Miller JK, McDougall S, Thomas S, Wiener J (2017). The impact of the brain-derived neurotrophic factor gene on trauma and spatial processing. J Clin Med.

[5] Ahmed AO, Mantini AM, Fridberg DJ, Buckley PF (2015). Brain-derived neurotrophic factor (BDNF) and neurocognitive deficits in people with schizophrenia: a meta-analysis. Psychiatry Res.

[6] Pillai A, Kale A, Joshi S, Naphade N, Raju MS, Nasrallah H, Mahadik SP (2010). Decreased BDNF levels in CSF of drug-naive first-episode psychotic subjects: correlation with plasma BDNF and psychopathology. Int J Neuropsychopharmacol.

[7] Rizos EN, Papadopoulou A, Laskos E, Michalopoulou PG, Kastania A, Vasilopoulos D, Katsafouros K, Lykouras L (2010). Reduced serum BDNF levels in patients with chronic schizophrenic disorder in relapse, who were treated with typical or atypical antipsychotics. World J Biol Psychiatry.

[8] Xiao W, Ye F, Liu C, Tang X, Li J, Dong H, Sha W, Zhang X (2017). Cognitive impairment in first-episode drug-naive patients with schizophrenia: relationships with serum concentrations of brain-derived neurotrophic factor and glial cell line-derived neurotrophic factor. Prog Neuro-Psychopharmacol Biol Psychiatry.

[9] Nieto R, Kukuljan M, Silva H (2013). BDNF and schizophrenia: from neurodevelopment to neuronal plasticity, learning, and memory. Front Psychiatry.

[10] Guzowski JF, Lyford GL, Stevenson GD, Houston FP, McGaugh JL, Worley PF, Barnes CA (2000). Inhibition of activity-dependent arc protein expression in the rat hippocampus impairs the maintenance of long-term potentiation and the consolidation of long-term memory. J Neurosci.

[11] Gururajan A, Hill RA, van den Buuse M (2015). Brain-derived neurotrophic factor heterozygous mutant rats show selective cognitive changes and vulnerability to chronic corticosterone treatment. Neuroscience..

[12] Zhang XY, Chen DC, Xiu MH, Haile CN, Luo X, Xu K, Zhang HP, Zuo L, Zhang Z, Zhang X, Kosten TA, Kosten TR (2012). Cognitive and serum BDNF correlates of BDNF Val66Met gene polymorphism in patients with schizophrenia and normal controls. Hum Genet.

[13] Zhang XY, Liang J, Chen DC, Xiu MH, Yang FD, Kosten TA, Kosten TR (2012). Low BDNF is associated with cognitive impairment in chronic patients with schizophrenia. Psychopharmacology (Berl).

[14] Gerlai R, McNamara A, Choi-Lundberg DL, Armanini M, Ross J, Powell-Braxton L, Phillips HS (2001). Impaired water maze learning performance without altered dopaminergic function in mice heterozygous for the GDNF mutation. Eur J Neurosci.

[15] Niitsu T, Shirayama Y, Matsuzawa D, Shimizu E, Hashimoto K, Iyo M (2014). Association between serum levels of glial cell-line derived neurotrophic factor and attention deficits in schizophrenia. Neurosci Lett.

[16] Skibinska M, Kapelski P, Pawlak J, Rajewska-Rager A, Dmitrzak-Weglarz M, Szczepankiewicz A, Czerski P, Twarowska-Hauser J (2017). Glial cell line-derived neurotrophic factor (GDNF) serum level in women with schizophrenia and depression, correlation with clinical and metabolic parameters. Psychiatry Res.

[17] Tunca Z, Kivircik Akdede B, Ozerdem A, Alkin T, Polat S, Ceylan D, Bayin M, Cengizcetin Kocuk N, Simsek S, Resmi H (2015). Diverse glial cell line-derived neurotrophic factor (GDNF) support between mania and schizophrenia: a comparative study in four major psychiatric disorders. Eur Psychiatry.

[18] Kirkpatrick B, Mucci A, Galderisi S (2017). Primary, enduring negative symptoms: an update on research. Schizophr Bull.

[19] Akyol ES, Albayrak Y, Beyazyuz M, Aksoy N, Kuloglu M, Hashimoto K (2015). Decreased serum levels of brain-derived neurotrophic factor in schizophrenic patients with deficit syndrome. Neuropsychiatr Dis Treat.

[20] Valiente-Gomez A, Amann BL, Marmol F, Oliveira C, Messeguer A, Lafuente A, Pomarol-Clotet E, Bernardo AM (2014). Comparison of serum BDNF levels in deficit and nondeficit chronic schizophrenia and healthy controls. Psychiatry Res.

[21] Wang X, Yao SQ, Fan XH, Yi YQ, Zhu WA, Yi JY (2005). The Chinese version of the schedule for the deficit syndrome: reliability and validity. Chin J Clin Psychol.

[22] Mueser KT, Curran PJ, McHugo GJ (1997). Factor structure of the brief psychiatric rating scale in schizophrenia. Psychol Assess.

[23] Woods SW (2003). Chlorpromazine equivalent doses for the newer atypical antipsychotics. J Clin Psychiatry.

[24] Carpenter WT, Heinrichs DW, Wagman AM (1988). Deficit and nondeficit forms of schizophrenia: the concept. Am J Psychiatry.

[25] Cubeddu A, Bucci F, Giannini A, Russo M, Daino D, Russo N, Merlini S, Pluchino N, Valentino V, Casarosa E, Luisi S, Genazzani AR (2011). Brain-derived neurotrophic factor plasma variation during the different phases of the menstrual cycle in women with premenstrual syndrome. Psychoneuroendocrinology..

[26] Pan W, Banks WA, Fasold MB, Bluth J, Kastin AJ (1998). Transport of brain-derived neurotrophic factor across the blood-brain barrier. Neuropharmacology..

[27] Lai CY, Scarr E, Udawela M, Everall I, Chen WJ, Dean B (2016). Biomarkers in schizophrenia: a focus on blood based diagnostics and theranostics. World J Psychiatry.

[28] Iritani S, Niizato K, Nawa H, Ikeda K, Emson PC (2003). Immunohistochemical study of brain-derived neurotrophic factor and its receptor, TrkB, in the hippocampal formation of schizophrenic brains. Prog Neuro-Psychopharmacol Biol Psychiatry.

[29] Weickert CS, Hyde TM, Lipska BK, Herman MM, Weinberger DR, Kleinman JE (2003). Reduced brain-derived neurotrophic factor in prefrontal cortex of patients with schizophrenia. Mol Psychiatry.

[30] Martinotti G, Di Iorio G, Marini S, Ricci V, De Berardis D, Di Giannantonio M (2012). Nerve growth factor and brain-derived neurotrophic factor concentrations in schizophrenia: a review. J Biol Regul Homeost Agents.

[31] Xiao W, Ye F, Ma L, Tang X, Li J, Dong H, Sha W, Zhang X (2016). Atypical antipsychotic treatment increases glial cell line-derived neurotrophic factor serum levels in drug-free schizophrenic patients along with improvement of psychotic symptoms and therapeutic effects. Psychiatry Res.

[32] Shao Z, Dyck LE, Wang H, Li XM (2006). Antipsychotic drugs cause glial cell line-derived neurotrophic factor secretion from C6 glioma cells. J Psychiatry Neurosci.

[33] Rorick-Kehn LM, Johnson BG, Knitowski KM, Salhoff CR, Witkin JM, Perry KW, Griffey KI, Tizzano JP, Monn JA, McKinzie DL, Schoepp DD (2007). In vivo pharmacological characterization of the structurally novel, potent, selective mGlu2/3 receptor agonist LY404039 in animal models of psychiatric disorders. Psychopharmacology.

[34] Patil ST, Zhang L, Martenyi F, Lowe SL, Jackson KA, Andreev BV, Avedisova AS, Bardenstein LM, Gurovich IY, Morozova MA, Mosolov SN, Neznanov NG, Reznik AM, Smulevich AB, Tochilov VA, Johnson BG, Monn JA, Schoepp DD (2007). Activation of mGlu2/3 receptors as a new approach to treat schizophrenia: a randomized phase 2 clinical trial. Nat Med.

[35] Yu M, Tang X, Wang X, Zhang X, Zhang X, Sha W, Yao S, Shu N, Zhang X, Zhang Z (2015). Neurocognitive impairments in deficit and non-deficit schizophrenia and their relationships with symptom dimensions and other clinical variables. PLoS One.

[36] Niitsu T, Shirayama Y, Matsuzawa D, Hasegawa T, Kanahara N, Hashimoto T, Shiraishi T, Shiina A, Fukami G, Fujisaki M, Watanabe H, Nakazato M, Asano M, Kimura S, Hashimoto K, Iyo M (2011). Associations of serum brain-derived neurotrophic factor with cognitive impairments and negative symptoms in schizophrenia. Prog Neuro-Psychopharmacol Biol Psychiatry.

[37] Man L, Lv X, Du XD, Yin G, Zhu X, Zhang Y, Soares JC, Yang XN, Chen X, Zhang XY (2018). Cognitive impairments and low BDNF serum levels in first-episode drug-naive patients with schizophrenia. Psychiatry Res.

[38] Airaksinen MS, Saarma M (2002). The GDNF family: signalling, biological functions and therapeutic value. Nat Rev Neurosci.

[39] Lin PY, Tseng PT (2015). Decreased glial cell line-derived neurotrophic factor levels in patients with depression: a meta-analytic study. J Psychiatr Res.

[40] Naumenko VS, Kondaurova EM, Bazovkina DV, Tsybko AS, Ilchibaeva TV, Khotskin NV, Semenova AA, Popova NK (2014). Effect of GDNF on depressive-like behavior, spatial learning and key genes of the brain dopamine system in genetically predisposed to behavioral disorders mouse strains. Behav Brain Res.

[41] Nanobashvili A, Airaksinen MS, Kokaia M, Rossi J, Asztely F, Olofsdotter K, Mohapel P, Saarma M, Lindvall O, Kokaia Z (2000). Development and persistence of kindling epilepsy are impaired in mice lacking glial cell line-derived neurotrophic factor family receptor alpha 2. Proc Natl Acad Sci U S A.

[42] Carlino D, Leone E, Di Cola F, Baj G, Marin R, Dinelli G, Tongiorgi E, De Vanna M (2011). Low serum truncated-BDNF isoform correlates with higher cognitive impairment in schizophrenia. J Psychiatr Res.

[43] Cotman CW, Berchtold NC, Christie LA (2007). Exercise builds brain health: key roles of growth factor cascades and inflammation. Trends Neurosci.

[44] Zhang XY, Tan YL, Chen DC, Tan SP, Yang FD, Wu HE, Zunta-Soares GB, Huang XF, Kosten TR, Soares JC (2016). Interaction of BDNF with cytokines in chronic schizophrenia. Brain Behav Immun.

[45] Kastin AJ, Akerstrom V, Pan W (2003). Glial cell line-derived neurotrophic factor does not enter normal mouse brain. Neurosci Lett.

